# Stress-Induced Proliferation and Cell Cycle Plasticity of Intracellular *Trypanosoma cruzi* Amastigotes

**DOI:** 10.1128/mBio.00673-18

**Published:** 2018-07-10

**Authors:** Peter C. Dumoulin, Barbara A. Burleigh

**Affiliations:** a Department of Immunology and Infectious Diseases, Harvard T.H. Chan School of Public Health, Boston, Massachusetts, USA; University of Pittsburgh

**Keywords:** Chagas, amastigote, benznidazole, cell cycle, plasticity, stress

## Abstract

The mammalian stages of the parasite Trypanosoma cruzi, the causative agent of Chagas disease, exhibit a wide host species range and extensive within-host tissue distribution. These features, coupled with the ability of the parasites to persist for the lifetime of the host, suggest an inherent capacity to tolerate changing environments. To examine this potential, we studied proliferation and cell cycle dynamics of intracellular T. cruzi amastigotes experiencing transient metabolic perturbation or drug pressure in the context of an infected mammalian host cell. Parasite growth plasticity was evident and characterized by rapid and reversible suppression of amastigote proliferation in response to exogenous nutrient restriction or exposure to metabolic inhibitors that target glucose metabolism or mitochondrial respiration. In most instances, reduced parasite proliferation was accompanied by the accumulation of amastigote populations in the G_1_ phase of the cell cycle, in a manner that was rapidly and fully reversible upon release from the metabolic block. Acute amastigote cell cycle changes at the G_1_ stage were similarly observed following exposure to sublethal concentrations of the first-line therapy drug, benznidazole, and yet, unlike the results seen with inhibitors of metabolism, recovery from exposure occurred at rates inversely proportional to the concentration of benznidazole. Our results show that T. cruzi amastigote growth plasticity is an important aspect of parasite adaptation to stress, including drug pressure, and is an important consideration for growth-based drug screening.

## IMPORTANCE

Infection with the intracellular parasite Trypanosoma cruzi can cause debilitating and potentially life-threatening Chagas disease, where long-term parasite persistence is a critical determinant of clinical disease progression. Such tissue-resident T. cruzi amastigotes are refractory to immune-mediated clearance and to drug treatment, suggesting that in addition to exploiting immune avoidance mechanisms, amastigotes can facilitate their survival by adapting flexibly to diverse environmental stressors. We discovered that T. cruzi intracellular amastigotes exhibit growth plasticity as a strategy to adapt to and rebound from environmental stressors, including metabolic blockades, nutrient starvation, and sublethal exposure to the first-line therapy drug benznidazole. These findings have important implications for understanding parasite persistence, informing drug development, and interpreting drug efficacy.

## INTRODUCTION

The protozoan parasite Trypanosoma cruzi establishes lifelong infection in mammalian hosts, where it can colonize diverse cell and tissue types. Approximately 8 million people harbor chronic T. cruzi infection (1), which can lead to the development of an aggressive inflammatory cardiomyopathy and/or the gastrointestinal megasyndromes characteristic of Chagas disease (2). Despite the complex etiology of human Chagas disease, parasite persistence is recognized as a key determinant underlying the development of clinical disease (3–5). The mechanistic basis for T. cruzi persistence is not well understood (6) but is the subject of increased scrutiny given that the parasite is refractory to killing by the available antitrypanosomals in the chronic stage of infection (7, 8).

T. cruzi colonizes diverse tissues in a broad range of mammalian hosts (9, 10), where its replication is entirely intracellular. Host cell infection is established by motile extracellular trypomastigotes that invade both immune and nonimmune cell types and then convert to the amastigote form, which replicates in the host cytoplasm (11). Within a single lytic cycle, amastigotes complete several rounds of division by binary fission and convert to trypomastigotes, which rupture the host cell membrane to allow dissemination of the parasite. With its broad host range (12, 13) and propensity to infect a variety of cell types *in vivo* and *in vitro* (9, 10), T. cruzi has the capacity to survive and proliferate in diverse metabolic environments and under conditions of various nutrient availabilities. Such predicted metabolic flexibility likely plays an integral role in the overall mechanism governing tissue persistence of T. cruzi.

The growth potential of a T. cruzi amastigote, as an obligate intracellular parasite, is intimately coupled to its host cellular metabolic machinery (14–16). The carbon sources fueling amastigote energy demands or anabolic processes are predicted to be diverse (17–20). Recent studies demonstrated that isolated T. cruzi amastigotes can utilize glucose or glutamine to fuel energy-generating processes and that intracellular amastigotes take up glucose (15) and triacylglycerides (21) from their host cells. Additionally, the availability of these nutrient sources influences parasite growth (15, 21). Despite this recent knowledge, the true potential for T. cruzi amastigotes to respond dynamically to environmental challenges, and their mechanisms, remains unknown.

Many unicellular organisms respond to changes in nutrient availability by adjusting their growth rates through changes in cell cycle dynamics (22). For instance, the extracellularly dividing stages of *Leishmania* parasites accumulate in the G_1/0_ phase in the absence of exogenous purines or fetal bovine serum (FBS) (23, 24). To examine the potential for growth plasticity in the intracellular T. cruzi amastigotes, we assessed the responses of cultured parasites to a range of inhibitory conditions, including metabolic blockers and benznidazole (Bz), which, despite its status as the first-line therapeutic agent for Chagas disease, has marginal clinical effectiveness (25). Presently, we found that T. cruzi amastigotes dramatically modulate their proliferation rates, as needed, from barely perceptible to rapid growth and that this modulation is enabled by reversible accumulation within and release from the cell cycle G_1_ phase. These processes occur within a single lytic cycle, providing the first experimental evidence that T. cruzi amastigotes are poised to respond to their environment in ways predicted to contribute to the resilience of this successful pathogen.

## RESULTS

### Exogenous nutrient restriction reversibly slows proliferation of intracellular T. cruzi amastigotes.

**FIG 1:**
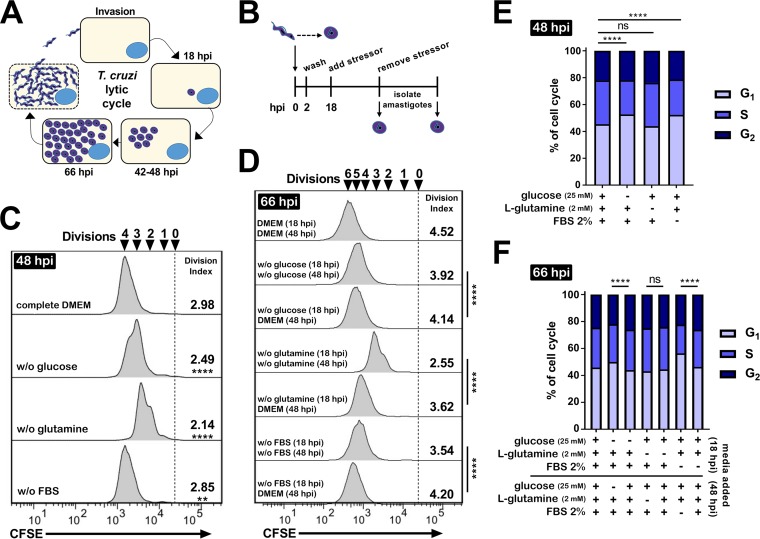
T. cruzi amastigotes respond to exogenous nutrient availability by altering proliferation and cell cycle. (A) Schematic of unabated invasion, differentiation, and growth of T. cruzi Tula-βgal *in vitro*. (B) Experimental design used to measure responsiveness of T. cruzi amastigote growth *in vitro*. (C and D) Flow cytometry histograms of amastigotes (CFSE) isolated at 48 hpi (C) or 66 hpi (D) under the indicated growth conditions. Division indices are compared using one-way ANOVA and a *post hoc* Dunnett’s test for multiple comparisons (****, *P* < 0.0001; **, *P* < 0.01; ns, not significant). (E and F) Cell cycle profiles for T. cruzi amastigotes isolated from infected HFF at 48 hpi (E) or 66 hpi (F) under the indicated exogenous starvation conditions initiated at 18 hpi. Comparisons were made using a chi-squared test (G_1_ versus S/G_2_) and Bonferroni correction for multiple testing.

T. cruzi amastigotes proliferate via progressive rounds of binary fission in mammalian host cells before conversion to motile trypomastigotes, which exit the host cell and disseminate infection in a lytic cycle (Fig. 1A). Recent studies have begun to identify extrinsic factors that impinge on the replicative capacity of intracellular T. cruzi amastigotes (14, 15, 21), but little is known regarding the level of flexibility that these parasites exhibit in response to changes in their immediate environment. To approach this issue, we first examined changes in intracellular T. cruzi growth rates and cell cycle profiles in response to transient nutrient restriction. The T. cruzi Tulahuén β-galactosidase (Tula-βgal) strain (26) often used in high-throughput screens (14, 27, 28) was used for these studies. The intracellular replicative phase of this parasite strain typically extends from 18 to ~72 h postinfection (hpi) *in vitro*, providing a window to examine amastigote proliferation dynamics within a single lytic cycle (Fig. 1A and B). Human foreskin fibroblasts (HFF) were selected as host cells given their capacity to tolerate nutrient deprivation (see Fig. S1A in the supplemental material) (29).

10.1128/mBio.00673-18.1 FIG S1  Exogenous nutrient restriction impedes amastigote growth. (A and B) CellTiter-Fluor (host cell) fluorescence (A) and Beta-Glo luminescence (parasite) (B) measured at 66 hpi under the indicated starvation conditions begun at 18 hpi (*n* = 16 per condition). Means ± SD are shown. Significance data were determined using one-way ANOVA and a *post hoc* Dunnett’s multiple-comparison test for complete medium (****, *P* < 0.0001; ***, *P* < 0.001; **, *P* < 0.01; *, *P* < 0.05). (C) Amastigotes per infected cell at 66 hpi (*n* = 40 per condition); medians are indicated in red. A Kruskal-Wallis test was used to determine significance, and a Dunn’s *post hoc* test was used for individual comparisons (*, *P* < 0.05; ****, *P* < 0.0001). Download FIG S1, TIF file, 1.1 MB. Copyright © 2018 Dumoulin and Burleigh. 2018 Dumoulin and Burleigh This content is distributed under the terms of the Creative Commons Attribution 4.0 International license.

Intracellular T. cruzi infection of HFF was established under nutrient-replete conditions (Dulbecco’s modified Eagle medium [DMEM] containing 25 mM glucose, 2 mM glutamine, and 2% heat-inactivated FBS), and infected cultures were then shifted to medium lacking glucose, glutamine, or FBS at 18 hpi, prior to the first division of the fully differentiated amastigotes (Fig. 1A and B). Restriction of these exogenous supplements, individually, reduced overall parasite load (Fig. S1B), with reduced numbers of intracellular amastigotes per infected cell (Fig. S1C). We then utilized a flow cytometric assay to establish whether these differences corresponded to a reduction in amastigote proliferation as measured by the average number of parasite cell divisions (division index) achieved at the indicated times (Fig. 1C and D). Consistent with a reduced rate of replication, we found modest but statistically significant changes in amastigote proliferation at 48 hpi (Fig. 1C) and 66 hpi (Fig. 1D) following restriction of glucose, glutamine, or FBS beginning at 18 hpi. Reintroduction of nutrient-replete medium (at 48 hpi) was associated with a relative increase in intracellular amastigote proliferation (measured at 66 hpi) compared to cultures that were refreshed with medium depleted for specific nutrients (Fig. 1D). Cell cycle profiles determined for intracellular amastigotes (Fig. S2) revealed that restriction of exogenous glucose or FBS availability resulted in a greater proportion of the parasite population in the G_1_ phase (Fig. 1E). Upon a return to nutrient-replete medium, the population reverted, with cell cycle phase proportions similar to those of untreated controls (Fig. 1F). Glutamine restriction was the exception, where, despite its greater relative impact upon amastigote proliferation (Fig. 1C and D), concomitant changes in the cell cycle profile were not seen (Fig. 1E and F).

10.1128/mBio.00673-18.2 FIG S2  Cell cycle profiling of isolated amastigote populations. A schematic of amastigote isolation (top) and representative models generated from raw data using individual amastigotes stained with DAPI (middle) are shown. A normalized (to 100%) representation of cell cycle models is shown (bottom). Download FIG S2, TIF file, 1.9 MB. Copyright © 2018 Dumoulin and Burleigh. 2018 Dumoulin and Burleigh This content is distributed under the terms of the Creative Commons Attribution 4.0 International license.

An independent T. cruzi isolate (CL Brener) was also tested for its response to glucose deprivation, which yielded results similar to those generated with the Tula-βgal strain. CL Brener amastigotes have a longer doubling time than Tula-βgal (Fig. S3A versus Fig. 1) and maintain a greater fraction of the parasite population in the G_1_ phase of the cell cycle (~52% to 66% for CL Brener [see Fig. S3B and D] versus 45% for Tula-βgal at 48 hpi [Fig. 1E]). This property extended to two subpopulations of CL Brener where the slower-growing population contained a greater proportion of G_1_ parasites (Fig. S3C to E). Thus, in addition to intrinsic cell cycle regulation, variable intracellular amastigote growth rates, between or within different parasite isolates, may be due in part to delayed progression through a G_1_ checkpoint, rather than to a universal slowing of all phases of the cell cycle. In both T. cruzi strains, the intracellular amastigotes continued to proliferate during exogenous nutrient restriction, indicating that the increased proportion of parasites occupying the G_1_ phase of the cell cycle represented accumulation at this phase rather than exit from the cell cycle (i.e., G_0_). Thus, despite modest impacts of transient carbon source restriction on intracellular T. cruzi growth, these data provide evidence that amastigote proliferation and cell cycle dynamics are responsive to environmental changes within the time frame of a single lytic cycle at the population level. However, this amastigote growth plasticity is not exclusively determined at G_1_ to S, as indicated by the results seen with glutamine restriction and phase transition, and therefore it is likely that distinct mechanisms govern parasite proliferation in response to specific metabolic cues.

10.1128/mBio.00673-18.3 FIG S3  Characterization of the proliferation of the CL Brener isolate and its response to glucose restriction. (A) Flow cytometry histograms of isolated CL Brener amastigotes (CFSE) collected at 48 hpi following changes in the media at 18 hpi. Division indices are compared using a *t* test (****, *P* < 0.0001). (B) Cell cycle distributions of CL Brener amastigotes collected at 48 hpi following changes to the medium at 18 hpi. Comparisons were made using a chi-squared test (G_1_ versus S/G_2_). (C) Histograms of CL Brener amastigotes (CFSE) isolated at the indicated time points. (D) Cell cycle distributions of CL Brener amastigotes at the given time points. (E) Cell cycle distributions of CL Brener amastigotes based on proliferation (population A versus population B) as measured by CFSE at the indicated time points. Download FIG S3, TIF file, 2.4 MB. Copyright © 2018 Dumoulin and Burleigh. 2018 Dumoulin and Burleigh This content is distributed under the terms of the Creative Commons Attribution 4.0 International license.

### Targeted inhibition of T. cruzi amastigote metabolism is cytostatic and leads to accumulation of amastigotes in G_1_.

**FIG 2:**
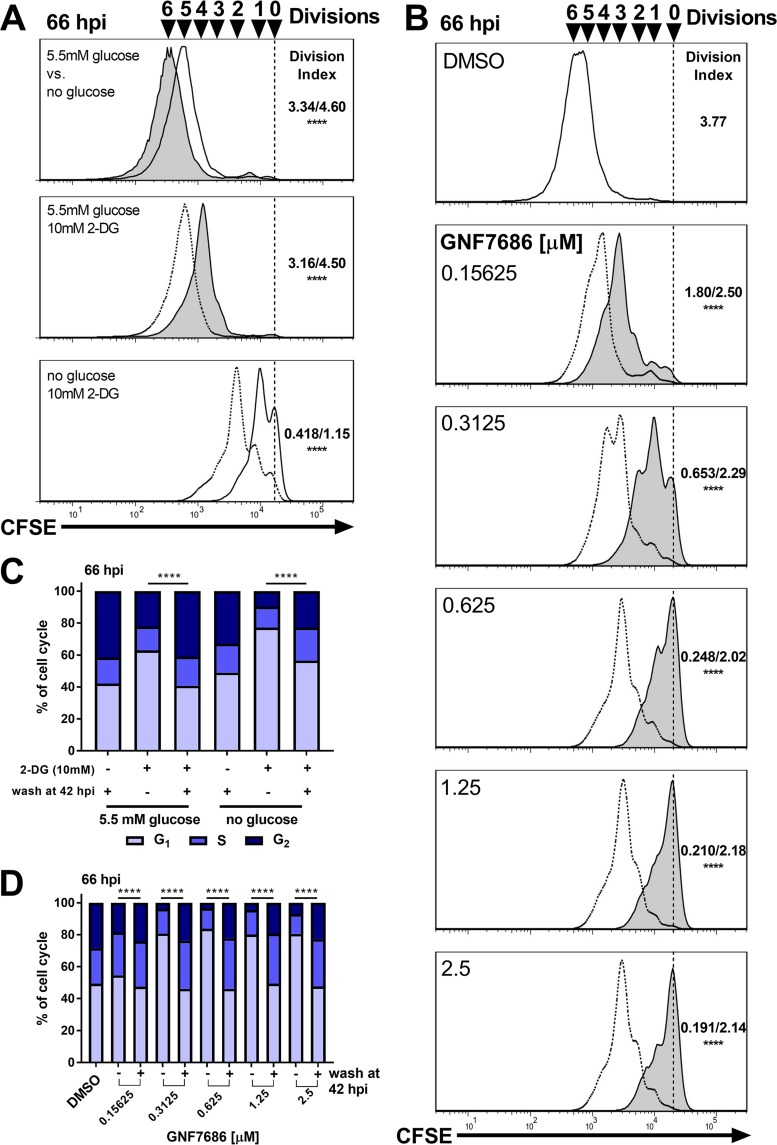
Rapid recovery of T. cruzi amastigote growth and cell cycle phase distribution following exposure to metabolic inhibitors. (A) Flow cytometry histograms of amastigotes (CFSE) isolated at 66 hpi. Cultures grown in 5.5 mM glucose are indicated with a solid line and filled histogram and cultures grown in medium without glucose are shown with a solid line and unfilled histograms. Cultures washed at 42 hpi are shown with a dotted line. Division indices (no glucose/5.5 mM glucose or not washed/washed) are compared using one-way ANOVA and a *post hoc* Dunnett’s multiple-comparison test where indicated (****, *P* < 0.0001). (B) Flow cytometry histograms of amastigotes (CFSE) isolated at 66 hpi. Histograms from cultures washed at 42 hpi are indicated with a dotted line. Division indices (not washed/washed) are compared using one-way ANOVA and a *post hoc* Dunnett’s multiple-comparison test (****, *P* < 0.0001). (C and D) Cell cycle distribution of amastigotes at 66 hpi under the indicated conditions with 2-DG (C) or GNF7686 (D). Comparisons were made using a chi-squared test (G_1_ versus S/G_2_) with Bonferroni correction for multiple testing.

Intracellular T. cruzi amastigotes are potentially buffered from the effects of exogenous nutrient restriction by the ability to access residual or alternative nutrient pools in the host cell. Therefore, we examined amastigote growth and cell cycle dynamics in response to more-pronounced metabolic inhibition. We utilized 2-deoxyglucose (2-DG), which targets glucose metabolism in the parasite and the host cell, and the small molecule inhibitor GNF7686 (30), which has been shown to selectively block mitochondrial respiration in intracellular T. cruzi amastigotes (15). Consistent with previous results (15, 30), we found that 2-DG and GNF7686 inhibit intracellular amastigote growth in a concentration-dependent manner (Fig. S4A and B) where the inhibitory effect of 2-DG is potentiated by the absence of glucose (Fig. S4A) (15). Microscopic examination of infected cells supported this finding (Fig. S4C and D) and revealed the continued presence of intracellular amastigotes following exposure to high concentrations of 2-DG (10 mM) or GNF7686 (2.5 µM). Under these conditions, amastigotes display the typical kinetoplast and nuclear morphology expected for healthy amastigotes (Fig. S4G) (dimethyl sulfoxide [DMSO]) as opposed to the morphology of parasites dying from ketoconazole exposure (31) (Fig. S4G). These treatments resulted in severe restriction of amastigote proliferation, particularly at higher GNF7686 concentrations or with 2-DG in glucose-free medium, by 42 hpi (Fig. S5A and B). Parasite populations displaying reduced proliferation rates in response to these metabolic blockades contained a greater proportion of amastigotes in the G_1_ phase of the cell cycle (Fig. S5C and D). To examine the plasticity of this growth inhibition response, washout experiments were performed in which parasite-infected cultures were treated from 18 to 42 hpi with 10 mM 2-DG or a range of GNF7686 concentrations, followed by removal of inhibitor at 42 hpi, and amastigote proliferation and cell cycle profiles were evaluated at 66 hpi (Fig. 2). Upon washout of these inhibitors, we observed a robust rebound in parasite proliferation (Fig. 2A and B) and a progression of amastigotes into S phase in greater proportions (Fig. 2C and D) that were indicative of recovery. As noted above for nutrient restriction (Fig. 1), intracellular amastigotes continued to proliferate even when subjected to high concentrations of 2-DG (10 mM) or GNF7686 at a >99% inhibitory concentration (>IC_99_) (Fig. 2B).

10.1128/mBio.00673-18.4 FIG S4  Inhibition of glycolysis or mitochondrial respiration suppresses amastigote growth. (A) Titration of 2-DG at 18 hpi in the indicated media and the resulting Beta-Glo luminescence (parasite, filled) and CellTiter-Fluor fluorescence (host cell, open) at 66 hpi. Means ± SD are shown (*n* = 4 per point). (B) Titration of GNF7686 at 18 hpi and the resulting Beta-Glo luminescence (parasite, filled) and CellTiter-Fluor fluorescence (host cell, open) at 66 hpi. Means ± SD are shown (*n* = 4 per point). (C and D) Amastigotes per infected cell counted at 42 hpi under conditions corresponding to the indicated glucose/2-DG concentrations (C) or GNF7686 concentrations (D). (E and F) Cultures washed at 42 hpi and counted at 66 hpi after exposure to 2-DG (E) or GNF7686 (F) at 18 hpi. A Kruskal-Wallis test was used to determine significance, and a Dunn’s *post hoc* test was used for individual comparisons (*, *P* < 0.05; **, *P* < 0.01; ***, *P* < 0.001; ****, *P* < 0.0001). (G) Representative images (DAPI staining) of amastigotes at the indicated time points (20 µM scale bar indicated). Insets: 4× zoomed sections of boxed area. Download FIG S4, TIF file, 2.8 MB. Copyright © 2018 Dumoulin and Burleigh. 2018 Dumoulin and Burleigh This content is distributed under the terms of the Creative Commons Attribution 4.0 International license.

10.1128/mBio.00673-18.5 FIG S5  Dose-dependent inhibition of amastigote growth using metabolic inhibitors. (A) Flow cytometry histograms of amastigotes (CFSE) isolated at 42 hpi under the indicated growth conditions (initiated at 18 hpi) with or without 10 mM 2-DG. Division indices were compared using a one-way ANOVA and a *post hoc* Dunnett’s multiple-comparison test (****, *P* < 0.0001). (B) Flow cytometry histograms of amastigotes (CFSE) isolated at 42 hpi following exposure to the indicated concentration of GNF7686 at 18 hpi. Division indices were compared using a one-way ANOVA and a *post hoc* Dunnett’s multiple-comparison test (****, *P* < 0.0001). (C) Cell cycle distribution of amastigotes at 42 hpi under the indicated conditions. (D) Cell cycle distribution of amastigotes at 42 hpi following addition of GNF7686 at the indicated concentrations. Comparisons were made to complete medium using a chi-squared test (G_1_ versus S/G_2_) and Bonferroni correction for multiple testing. Download FIG S5, TIF file, 0.7 MB. Copyright © 2018 Dumoulin and Burleigh. 2018 Dumoulin and Burleigh This content is distributed under the terms of the Creative Commons Attribution 4.0 International license.

**FIG 3:**
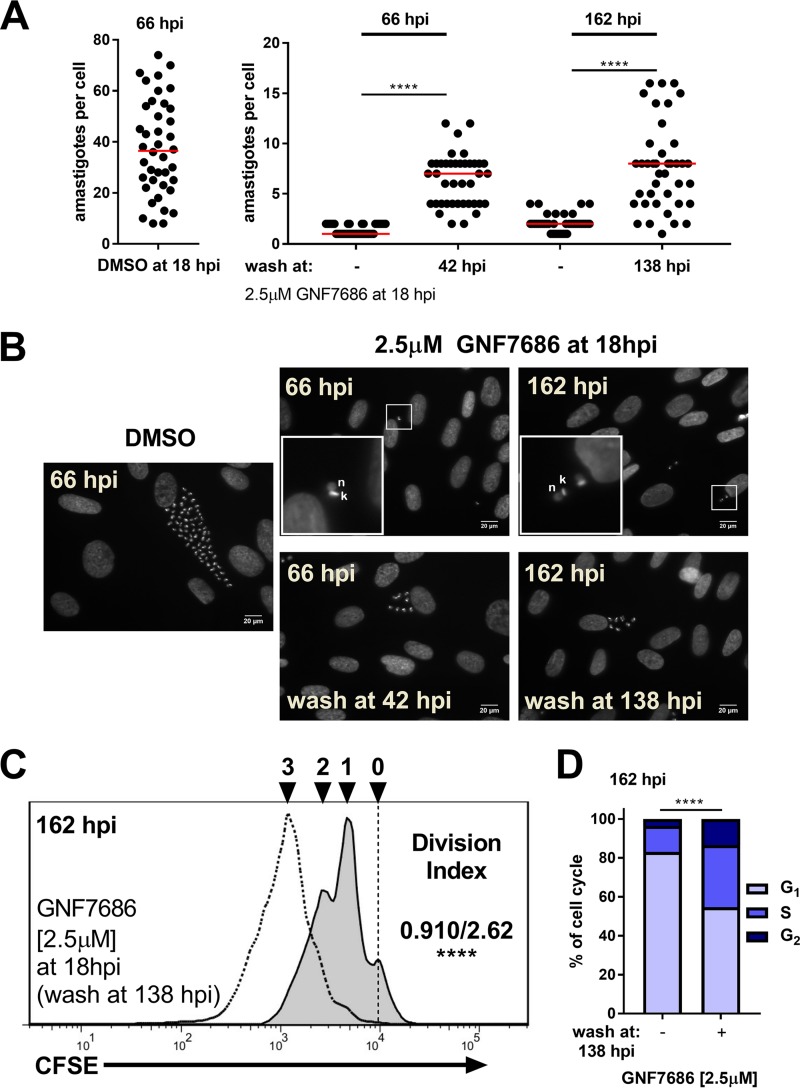
Intracellular T. cruzi amastigotes tolerate prolonged complex III inhibition. (A) Amastigotes per infected cell from coverslips collected at the indicated time points following addition of GNF7686 (2.5 µM) or DMSO at 18 hpi. Medians are shown in red (*n* = 40 per condition). A Kruskal-Wallis test was used to determine significance, and a Dunn’s *post hoc* test was used for individual comparisons (****, *P* < 0.0001). (B) Representative images (DAPI staining) of amastigotes at the indicated time points (20 µM scale bar indicated). Inset: 4× zoomed sections where present (lower left). (C) Flow cytometry histograms of amastigotes (CFSE) isolated at 162 hpi following addition of 2.5 µM GNF7686 at 18 hpi. Histograms are from cultures with constant GNF7686 (solid line filled histogram) or washed at 138 hpi (dotted line no fill). Division indices (not wash/wash at 138 hpi) are compared using a *t* test (****, *P* < 0.0001). (D) Cell cycle distribution of amastigotes at 162 hpi under the indicated conditions. Comparisons were made using a chi-squared test (G_1_ versus S/G_2_) with Bonferroni correction for multiple testing.

The lack of host cell toxicity associated with GNF7686 treatment (Fig. S4B and G) (30) permitted testing the effects of longer exposure times on amastigote growth and cell cycle dynamics. After a continuous 5-day exposure of T. cruzi-infected monolayers to 2.5 µM GNF7686 (>IC_99_), intracellular amastigotes persisted, proliferating at a very low rate (average doubling time of >6 days, versus 8 to 12 h in untreated cultures) (Fig. 3A to C) where 80% of the parasite population was in the G_1_ phase of the cell cycle (Fig. 3D). Following washout of GNF7686, amastigotes proliferated rapidly (Fig. 3C) with a concomitant progression of the parasite population from G_1_ to S phase (Fig. 3D). Thus, prolonged inhibition of amastigote respiration appears to have had no lingering detrimental consequences given the population-level rebound in growth once GNF7686 was removed. Other metabolic processes, such as glycolysis, likely provide the energy needed to sustain parasite growth and division (15, 30). Consistent with this hypothesis, elimination of glucose from the growth medium potentiated the inhibitory activity of GNF7686, whereas no differences were observed when glutamine or FBS was withheld (Fig. S6), suggesting that glucose metabolism can partially compensate for inhibition of mitochondrial respiration in intracellular T. cruzi amastigotes. Thus, our use of metabolic inhibitors as tools to probe the growth response of intracellular T. cruzi amastigotes clearly demonstrated that these parasites can respond rapidly to changes in their immediate host cell environment by tuning their proliferation and cell cycle dynamics and can do so within the time frame of a single lytic cycle.

10.1128/mBio.00673-18.6 FIG S6  Inhibitory activity of GNF7686 is potentiated in the absence of glucose. Titration of GNF7686 at 18 hpi in the indicated media and resulting (A) Beta-Glo luminescence (parasite, filled) and (B) CellTiter-Fluor fluorescence (host cell, open) at 66 hpi are indicated. Means ± SD are shown (*n* = 4 per point). Download FIG S6, TIF file, 0.4 MB. Copyright © 2018 Dumoulin and Burleigh. 2018 Dumoulin and Burleigh This content is distributed under the terms of the Creative Commons Attribution 4.0 International license.

### T. cruzi amastigotes respond to benznidazole exposure through accumulation in G_1_ and exhibit a postantibiotic effect after drug removal.

**FIG 4:**
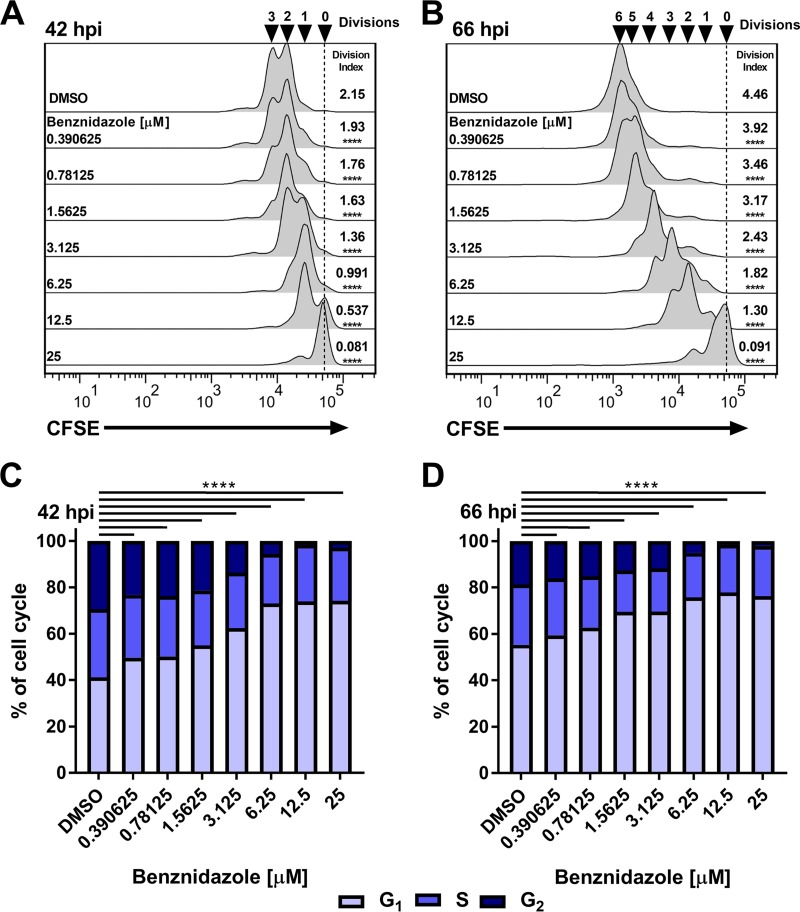
Acute benznidazole exposure drives intracellular T. cruzi amastigotes to accumulate in G_1_ and inhibits proliferation. (A and B) Flow cytometry histograms of isolated amastigotes (CFSE) at 42 hpi (A) and 66 hpi (B) following benznidazole treatment at 18 hpi. Division indices are compared to the results seen with DMSO using one-way ANOVA and a *post hoc* Dunnett’s multiple-comparison test for comparisons to DMSO data where indicated (****, *P* < 0.0001). (C and D) Amastigote cell cycle distributions at (C) 42 hpi and (D) 66 hpi following benznidazole treatment at 18 hpi. Comparisons were made using a chi-squared test (G_1_ versus S/G_2_) with Bonferroni correction for multiple testing.

**FIG 5:**
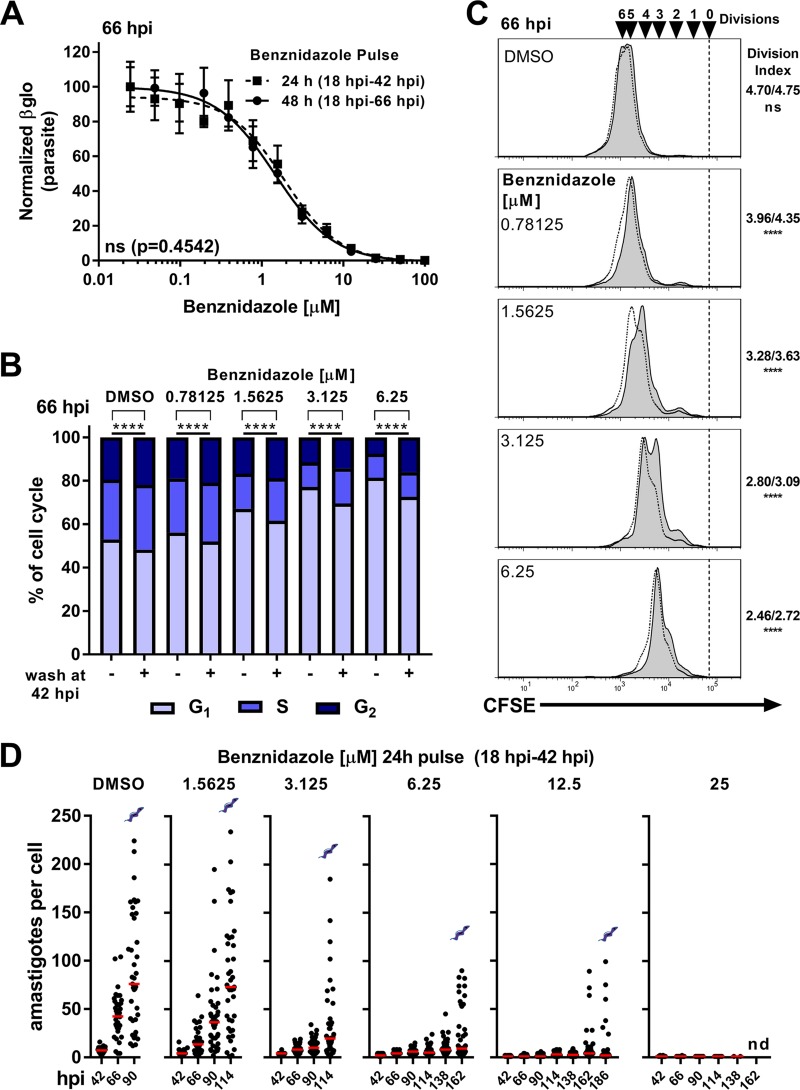
Recovery from acute benznidazole treatment is inversely proportional to the concentration of drug present during exposure. (A) Concentration-response relationship for the inhibitory effects of acute exposure to Bz for 24 h (filled squares) or 48 h (filled circles) on intracellular T. cruzi (Tula-βgal) amastigote growth as measured by Beta-Glo luminescence at 66 hpi. Means ± standard deviations (SD) are shown (*n* = 4 per point). (B) Cell cycle distribution of amastigotes at 66 hpi. Comparisons between washed or constant exposure to Bz were made using a chi-squared test (G_1_ versus S/G_2_) with Bonferroni correction for multiple testing. (C) Flow cytometry histogram overlays of isolated amastigotes (CFSE) at 66 hpi from cultures washed at 42 hpi (nonfilled histograms, dotted line) or without removal (filled histograms, solid line) of Bz. Division indices (no wash/wash at 42 hpi) were compared using a one-way ANOVA and a *post hoc* Dunnett’s multiple-comparison test (****, *P* < 0.0001). (D) Amastigotes per cell from coverslips collected at the indicated time points following a pulse of benznidazole (18 hpi to 42 hpi). Medians are indicated in red. Coverslips were not collected following detection of the presence of visible extracellular trypomastigotes (as indicated), which occurred in all cases except at the concentration of 25 µM (nd = none detected).

Perturbations such as redox imbalance and DNA damage can also trigger cell cycle changes in model organisms, and such stressors contribute to the mechanisms of action of many antimicrobial agents. The first-line treatment for Chagas disease, benznidazole (Bz), acts by inducing the formation of free radicals and electrophilic metabolites within the parasite (32) and yet sometimes fails to produce a sterilizing cure in humans (8). This observation raises the possibility that T. cruzi amastigotes can respond adaptively to Bz by regulating their proliferation to survive such treatment. We tested this possibility by examining the effects of Bz on T. cruzi amastigote growth and cell cycle distribution. Titration of Bz generated a typical *in vitro* drug sensitivity curve on the basis of total parasite luminescence (growth) (Fig. S7A) with derived IC_50_s similar to published values for the same parasite line (28). Acute Bz treatment resulted in concentration-dependent inhibition of amastigote proliferation as measured by flow cytometry (Fig. 4A and B) and microscopy (Fig. S7B and C) as well as in altered cell cycle profiles characterized by accumulation of parasites in the G_1_ phase (Fig. 4C and D). The presence of undivided amastigotes at both 42 and 66 hpi (Fig. 4A and B) suggests that Bz acts rapidly without altering the gross morphology of amastigote kinetoplasts and nuclei (Fig. S7D), indicating that the exposure had a cytostatic but not lethal effect. The inhibitory capacities of Bz were comparable in cultures that were pulsed with drug for 24 or 48 h (Fig. 5A) and consequently allowed us to study the ability of amastigotes to respond to this insult by using a 24-h pulse of drug. As shown (Fig. 5B and C), amastigotes recovered from a 24-h pulse of Bz (18 to 42 hpi) as evidenced by an increase in parasite proliferation measured at 66 hpi and an associated decrease in the level of amastigotes in G_1_ (Fig. 5B and C). However, unlike the rapid rebound that occurred following transient exposure to GNF7686 (Fig. 2B and D), the extent to which parasites rebounded from Bz exposure was found to be inversely proportional to the amount of drug present during the pulse (Fig. 5B and C), indicating that the concentration of Bz impacted subsequent growth following removal in a concentration-dependent manner, characteristic of a postantibiotic effect (33). Since amastigote cell cycle distribution and proliferation were still perturbed following removal of Bz, we investigated several possibilities to better understand amastigote growth plasticity under these conditions.

10.1128/mBio.00673-18.7 FIG S7  Benznidazole inhibits amastigote growth. (A) Titration of Bz at 18 hpi and resulting Beta-Glo luminescence (parasite, filled) and CellTiter-Fluor fluorescence (host cell, open) at 66 hpi. Means ± SD are shown (*n* = 4 per point). (B and C) Amastigotes per infected cell counted at 42 hpi (B) or 66 hpi (C) following exposure to Bz at the indicated concentration at 18 hpi. A Kruskal-Wallis test was used to determine significance, and a Dunn’s *post hoc* test was used for individual comparisons (*, *P* < 0.05; **, *P* < 0.01; ***, *P* < 0.001; ****, *P* < 0.0001). (D) Representative images (DAPI staining) of amastigotes at the indicated time points (20 µM scale bar indicated). Inset: 4× zoomed sections are presented (lower left). Download FIG S7, TIF file, 2.3 MB. Copyright © 2018 Dumoulin and Burleigh. 2018 Dumoulin and Burleigh This content is distributed under the terms of the Creative Commons Attribution 4.0 International license.

**FIG 6:**
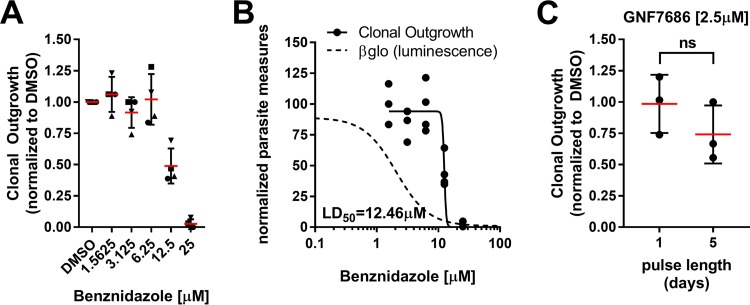
LD_50_ determination for benznidazole, 24-h pulse. (A) Clonal outgrowth (60 wells per plate, one plate per concentration for each independent replicate) following a 24-h pulse of Bz or DMSO followed by a 21-day recovery. Each experiment (indicated as squares, circles, triangles, or inverted triangles) is normalized to the number of clones seen under the DMSO condition from that given experiment (*n* = 4 experiments). Means ± SD are shown. (B) Clonal outgrowth determined on the basis of data from panel A (circles) graphed with the best-fit inhibitory curve (dotted line) and best-fit curve for clonal outgrowth (solid line). LD_50_ values were calculated using a best-fit curve for clonal outgrowth. (C) Clonal outgrowth normalized to DMSO treatments following either 1 day or 5 days of exposure to GNF7686 at a concentration of 2.5 µM (*n* = 3, 60 wells per independent replicate). Outgrowth data were compared using a *t* test (*P* = 0.267). Means ± SD are shown.

It is possible that the failure of amastigotes to return to an unperturbed replicative state was the result of damage accrued during Bz exposure (32, 34) that might result in eventual parasite death. Daily microscopic examination of infected cultures that received a 24-h pulse of Bz confirmed that parasites grew more slowly when pulsed with higher concentrations of Bz (Fig. 5D). On the basis of these data, we could not exclude the possibility that a portion of amastigotes succumb to Bz exposure and that the observed incomplete return to untreated proliferation rates (Fig. 5B and C and D) is simply a reflection of partial cytotoxicity and not necessarily of parasite plasticity. We therefore determined the 50% lethal acute dose (LD_50_) of Bz in relation to its IC_50_ by adapting a clonal outgrowth assay (35) following a 24-h drug pulse. Using this method, we determined that the LD_50_ of Bz (24-h pulse) was 12.46 µM, about nine times greater than the IC_50_ calculated for the same drug exposure time (Fig. 6A and B), and that, as anticipated, GNF7686 treatment at >IC_99_ did not result in parasite cytotoxicity (Fig. 6C). At a pulsed exposure to a 25 µM concentration, Bz clones could still be isolated in two of four experiments (Fig. 6A and B), indicating that under these conditions Bz is not 100% cytotoxic to intracellular T. cruzi amastigotes. To confirm this observation, we tested outgrowth of cultures infected at a multiplicity of infection (MOI) of 5 (i.e., nonclonal) and found that all wells produced trypomastigotes, indicating that this exposure is not completely sterilizing (Fig. S8A).

10.1128/mBio.00673-18.8 FIG S8  Benznidazole toxicity is not absolute but increases with exposure time and affects nondividing trypomastigotes. (A) Multiply infected cultures (MOI of 5) and time to first detection of extracellular trypomastigotes following a 24-h pulse of benznidazole (*n* = 12 per drug concentration). Means ± SD are shown. (B) Clonal outgrowth normalized to DMSO following exposure to various pulse lengths of benznidazole. (C) Numbers of motile trypomastigotes per well following a 24-h exposure to benznidazole or ketoconazole (*n* = 8 per condition). DMSO (gray filled circles), benznidazole (squares), and ketoconazole (filled triangles) are shown along with means ± SD. Download FIG S8, TIF file, 0.3 MB. Copyright © 2018 Dumoulin and Burleigh. 2018 Dumoulin and Burleigh This content is distributed under the terms of the Creative Commons Attribution 4.0 International license.

However, clinical use of Bz results in sustained serum levels due to a 12 h half-life in humans and to administration of multiple doses per day (36, 37). We therefore tested the impact of Bz pulse length at concentrations below the 24-h pulse LD_50_ but above the IC_50_, using clonal outgrowth to measure cytotoxicity (Fig. S8B). Prolonged (up to 5 days) exposure to Bz can increase cytotoxic potency, but such conditions were still unable to completely eliminate clonal outgrowth. Additionally, in line with the literature (34, 38), we found that Bz significantly altered trypomastigote motility at concentrations below the LD_50_, indicating that the nondividing cells were still susceptible to Bz (Fig. S8C). Thus, while growth of intracellular T. cruzi amastigotes is significantly slowed following transient Bz exposure (Fig. 4 and 5), the capacity of the parasite to rebound is reduced with greater exposure (Fig. 6; see also Fig. S8), indicating that both the level and time of exposure to this drug determine cytotoxicity.

## DISCUSSION

This study demonstrated the intrinsic capacity of intracellular Trypanosoma cruzi amastigotes to dynamically adjust their proliferation rates along with a correlated cell cycle in response to different stressors. Parasite growth plasticity was evident within a single lytic cycle following acute nutrient or metabolic stress and following exposure to sublethal doses of benznidazole, the first-line therapy for Chagas disease. The effect of transient nutrient withdrawal or chemical inhibition of glycolysis or respiration was characterized by a reversible slowing of intracellular amastigote proliferation accompanied by an increased proportion of parasites in the G_1_ phase of the cell cycle, in most instances. Simple nutrient withdrawal had a relatively mild effect on T. cruzi amastigote growth and cell cycle dynamics in the time frame analyzed. This was anticipated given the likely availability of alternative fuel sources in the host cell (39) and the potential for parasites to scavenge alternative carbon sources (15, 40, 41). In contrast, addition of 2-DG, which inhibits glycolysis in both the mammalian host cell and the parasite (15, 42), or inhibition of parasite respiration with GNF7686 (30) resulted in dramatic growth repression with a correspondingly large shift in the amastigote population to a G_1_ state. However, even with prolonged exposure to GNF7686, intracellular amastigotes continued to proliferate, albeit at a markedly reduced rate. The rebound from these metabolic blockades was rapid and apparently complete as indicated by proliferation assays and cell cycle profiles. These findings highlight the capacity of the intracellular T. cruzi amastigotes to vary their growth rates significantly, without exiting the cell cycle, and to remain poised to resume growth when conditions become more favorable.

We were surprised to find that inhibition of the mitochondrial electron transport chain in intracellular T. cruzi amastigotes using GNF7686 to inhibit cytochrome *b* was insufficient to kill parasites even after a 5-day exposure to the compound at >IC_99_. Clearly, amastigote growth was severely compromised, with doubling times increasing from 8 to 12 h to >6 days, but the parasites were still viable and slowly moving through their cell cycle. We postulate that energy generated via glycolysis is able to support amastigote survival and proliferation when parasite respiration is compromised, as indicated by the observation that glucose restriction sensitized amastigotes to GNF7686. While cytochrome *b* is a validated drug target for other pathogens (43), intracellular T. cruzi amastigotes appear to be adept at dealing with inhibition of the electron transport chain, likely negating mitochondrial respiration in T. cruzi as a drug target.

Selective progression through the cell cycle, mediated at the G_1_-to-S transition in response to stressors such as nutrient deprivation or DNA damage, is evident in many eukaryotes (44, 45); however, our data represent the first report that the intracellular amastigote forms of T. cruzi, the main life cycle stage targeted in drug development pipelines (27, 28, 30), differ in their proliferation rates in response to different stressors. The specific substrates and/or metabolites that are sensed by T. cruzi to allow progression through this G_1_ checkpoint have not been defined. Levels of AMP, free amino acids, and glucose are all well-studied correlates with a eukaryotic starvation response (46). T. cruzi has functional mTOR type signaling pathways to potentially translate these metabolite levels into a coordinated cellular response (47). In this light, the differential doubling times observed between T. cruzi isolates (48) highlighted here with the comparison of the Tulahuén and CL Brener strains, where slower doubling time correlated with an increased fraction of the amastigote population in the G_1_ phase of the cell cycle, suggest that the signals operating to initiate S phase, such as the necessary metabolite levels or their thresholds, differed between isolates. Such strain-specific properties influencing cell cycle progression may be an important determinant of tissue parasitism, drug efficacy, and pathogenesis.

Traditional measures of antitrypanosomal drug efficacy *in vitro* have relied on measurements of amastigote growth that cannot distinguish between amastigote death (cytocidal effects) and suppression of division (cytostatic effects) (49). Additionally, image-based screening methods set defined limits of detection for parasitized cells based on the number of amastigotes per cell to avoid false positives. These parameters may underestimate infection percentages and overestimate drug lethality when amastigote proliferation is suppressed to below the limits of detection (27, 28, 50). As a consequence, we found that the *in vitro* potency of the first-line therapy drug benznidazole may be overestimated. We observed a titratable, population-level effect on amastigote proliferation accompanied by an increased proportion of parasites in the G_1_ phase of the cell cycle. However, unlike the rapid rebounding of the entire amastigote population seen following other treatments, benznidazole-treated parasites return more slowly to a replicative state, the extent to which is inversely proportional to the amount of drug. We predict that such delayed recovery reflects macromolecular damage that inhibits cell cycle progression to allow repair and ensure the fidelity of replication (45). Among Chagas patients, following cessation of benznidazole treatment, a subset of individuals reverted from PCR-negative status to having detectable parasite DNA, indicating parasite persistence in such cases (8, 25). Several explanations have been proposed to account for this lack of sterilizing cure by benznidazole in humans, including inadequate biodistribution, low solubility, high levels of serum binding, and low bioavailability through extensive liver metabolism (51–54). On the basis of our current findings, it is conceivable that the flexibility of the T. cruzi amastigote may serve to protect tissue resident parasites from insufficient exposure to drug. At the same time, nondividing trypomastigotes are susceptible to the higher levels of Bz achievable in serum (34, 51), contributing to the PCR-negative status observed in many Chagas patients undergoing treatment, despite incomplete clearance of amastigotes from tissue.

In summary, our data highlight a previously unrecognized plasticity of T. cruzi amastigote growth rates in mammalian host cells. This endogenous capacity results in non-genetically determined cell cycle resistance to candidate pharmacotherapies. These dynamics act at a population level and can lead to greatly increased parasite doubling times without complete growth arrest. These processes are clearly distinct from spontaneous dormancy (55) and predict a sophisticated sensing and response pathway coupled to cell cycle regulation as an important route to long-term persistence of T. cruzi in mammalian hosts.

## MATERIALS AND METHODS

### Mammalian cell culture.

Mammalian cells were cultured in Dulbecco’s modified Eagle medium (DMEM; HyClone) supplemented with final concentrations of 25 mM glucose, 2 mM l-glutamine (Gibco), 100 U/ml penicillin-streptomycin (Gibco), and 10% FBS (Gibco) (DMEM-10). Cells infected with Trypanosoma cruzi were maintained in complete DMEM containing 2% FBS (DMEM-2) unless otherwise indicated. Cultures were maintained at 37°C in a 5% CO_2_ incubator.

### Parasite maintenance and preparation of trypomastigotes.

Tulahuén LacZ clone C4 (26) (ATCC, PRA-330) (Tula-βgal) was obtained directly from the ATCC and maintained by weekly passage in LLC-MK_2_ (ATCC, CCL-7) cells. Trypomastigotes were collected from culture supernatants that were centrifuged at 230 × *g* for 10 min to pellet any host cells. The resulting supernatant was collected, and trypomastigotes were pelleted at 2,060 × *g* for 10 min. Highly motile trypomastigotes were allowed to swim out of the pellet for ≥2 h at 37°C in a 5% CO_2_ incubator prior to collection and washing in DMEM-2. Purified trypomastigotes were suspended in DMEM-2 and allowed to infect subconfluent monolayers of HFF (kindly provided by S. Lourido, Whitehead Institute) for 2 h. The remaining extracellular parasites were removed with two phosphate-buffered saline (PBS) washes, fresh DMEM-2 was added to infected cultures, and cells were incubated as indicated.

### CFSE staining of trypomastigotes and amastigote isolation.

Purified trypomastigotes were resuspended in PBS at a concentration of 5 × 10^5^ trypomastigotes/ml and stained with carboxyfluorescein succinimidyl ester (CFSE; Thermo Fisher) at a final concentration of 1 µM for 15 min at 37°C. Staining solution was quenched with DMEM-2, and parasites were spun immediately at 2,060 × *g* for 10 min. The resulting pellet was resuspended in fresh DMEM-2. Stained trypomastigotes were allowed to infect for 2 h and washed twice prior to addition of fresh DMEM-2. At the indicated time points postinfection, monolayers were washed once with PBS and subjected to mild trypsinization (0.05% trypsin–EDTA; Gibco) to release cells from the tissue culture plastic. Once in suspension, cells were collected and spun at 300 × *g* for 10 min and washed once in PBS. The resulting cell pellets were resuspended in 500 µl PBS and lysed by passage through a 28-gauge needle 20 times. Lysates containing intact amastigotes were fixed with a final concentration of 1% paraformaldehyde (PFA)–PBS on ice for 20 min. Amastigotes were spun at 4,000 × *g* for 10 min immediately after fixation, and the resulting pellet was resuspended in 500 µl PBS and stored at 4°C until staining and acquisition were performed. This technique relies on dilution of the stain CFSE and provides a history of divisions that have been completed prior to host cell lysis and parasite isolation. As amastigotes divide, their progeny contribute exponentially greater numbers of detectable events; therefore, we utilized a model that normalizes these populations to generate a measure of the average number of divisions that an original amastigote has undergone, termed the division index (56).

### DNA staining and flow cytometry.

Directly prior to acquisition, amastigotes were pelleted at 4,000 × *g* for 10 min and resuspended in a staining solution of 0.1% Triton X-100–PBS containing 10 ng/ml DAPI (4′,6-diamidino-2-phenylindole) (Sigma-Aldrich). At least 10,000 events in the final amastigote gate were acquired per sample using an LSRII instrument (Becton Dickinson). Results were analyzed using FlowJo (Tree Star) proliferation and cell cycle modeling. Proliferation models were based on the CFSE intensity of samples collected at 18 hpi (undivided) for each experiment.

### Multiplex quantification of host cell viability and parasite luminescence.

Multiplex assays were modified slightly from previous work (14). At 1 day prior to infection, HFFs were trypsinized and seeded in 384-well plates (Corning) at a density of 1,500 cells per well in 30 µl DMEM-10. For infection, 10 µl of purified trypomastigotes per well was added at a multiplicity of infection of 1.25. At 2 h postinfection (hpi), plates were washed twice with PBS and replaced with 30 µl fresh DMEM-2 without phenol red. At 66 hpi, medium was removed and 10 µl CellTiter-Fluor (Promega) was added per well. Fluorescence was measured using an EnVision plate reader (PerkinElmer). Subsequently, 10 µl Beta-Glo (Promega) was added per well and luminescence was measured using an EnVision plate reader (PerkinElmer) following a 30-min incubation at room temperature. Background luminescence values from uninfected wells were subtracted from the values determined for infected wells to account for luminescence specific to host cells.

### Drug stocks.

Stock concentrations of 20 mM benznidazole (Sigma-Aldrich), 2 mM ketoconazole (Sigma-Aldrich), and 5 mM GNF7686 (Vitas-M Laboratory) were prepared by solubilization in DMSO. 2-Deoxy-d-glucose (Sigma-Aldrich) was resuspended in water to a stock concentration of 2 M.

### Fluorescence microscopy.

Cells were seeded at a density of 5 × 10^4^ cells per well on coverslips (EMD) in 24-well plates. At the indicated times postinfection, coverslips were fixed with 1% PFA–PBS. Prior to staining, coverslips were washed twice with PBS and subsequently stained with a 0.1% Triton X-100–PBS solution containing 100 ng/ml DAPI (Sigma-Aldrich) for 5 min at room temperature. Immediately after staining, coverslips were washed four times with PBS and mounted using ProLong Antifade (Thermo Fisher).

### Clonal outgrowth assays.

At 1 day prior to infection, 1 × 10^4^ HFFs per well were seeded in a 96-well plate. Approximately 25 purified trypomastigotes were added per well to allow approximately 50% of wells to become infected. Peripheral wells were not used due to the potential dehydration (60 internal wells per plate were used). One plate was used per drug concentration per replicate. At 2 hpi, wells were washed twice with 200 µl PBS per well. Benznidazole was added at 18 hpi. At the indicated times (24-h pulse of drug or longer), wells were washed twice with PBS and 200 µl DMEM-2 was added per well. Wells were visualized at 21 days postinfection (dpi) to reveal the presence of extracellular trypomastigotes.

### Statistics.

Comparisons from microscopy counts (number of amastigotes per infected cell) were made using a Kruskal-Wallis test (nonparametric) for significance and a Dunn’s *post hoc* test for individual comparisons. Division indices (levels of proliferation measured by flow cytometry) were compared using a one-way analysis of variance (ANOVA) for significance and Dunnett’s *post hoc* test for individual comparisons (56). Cell cycle phase distributions were compared using a chi-squared test (G_1_ versus S/G_2_), and Bonferroni correction was used to adjust *P* values for multiple testing. Differences in calculated IC_50_ values were assessed using an extra-sum-of-squares *F* test.
