# Supplementary figures and images for: Stress-Induced Proliferation and Cell Cycle Plasticity of Intracellular Trypanosoma cruzi Amastigotes
Source: mBio. 2018 Jul 10;9(4):e00673-18. doi: 10.1128/mBio.00673-18 (PMC6050952; doi:10.1128/mBio.00673-18)

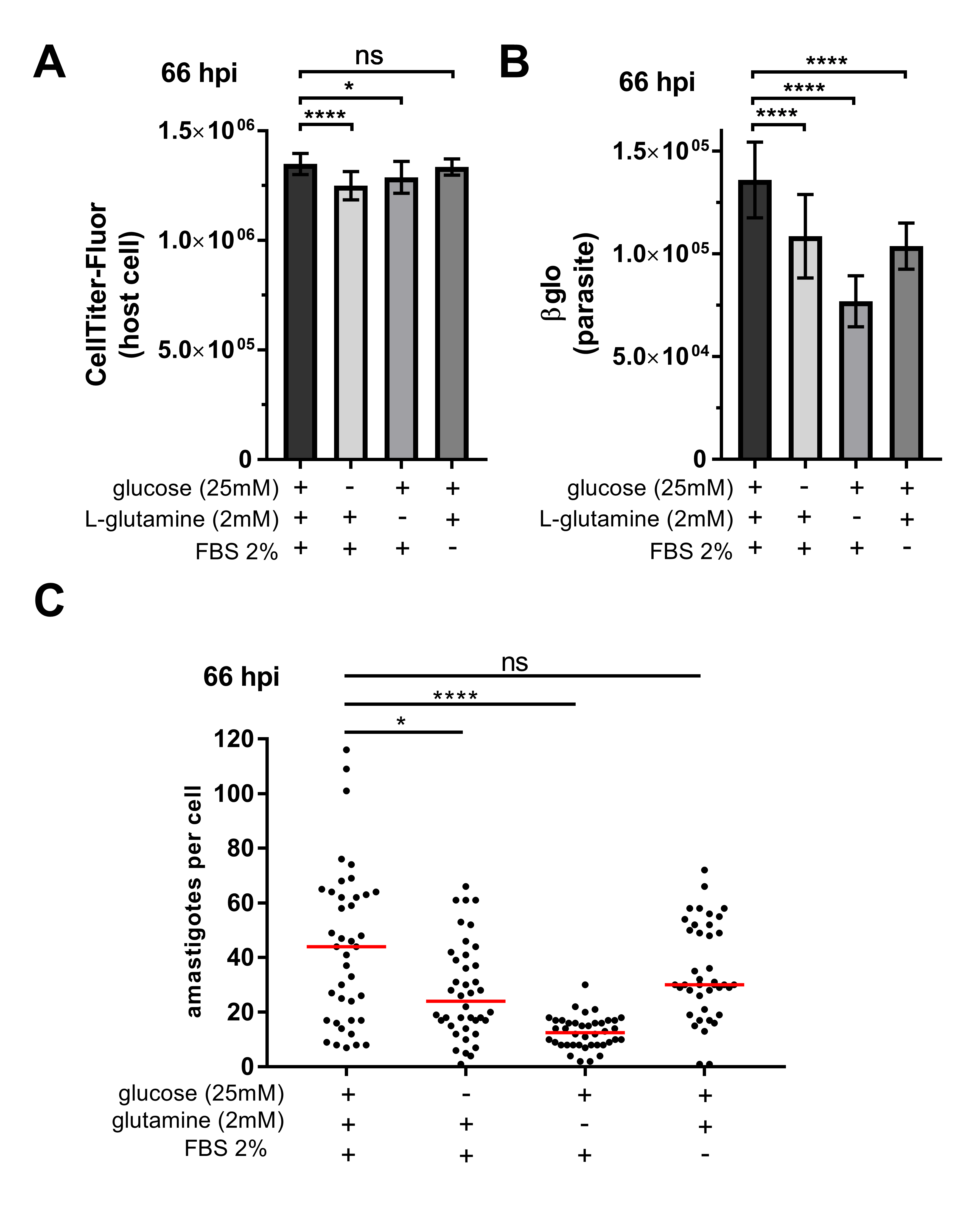

Supplement: FIG S1 [file mbo004183981sf1.tif]

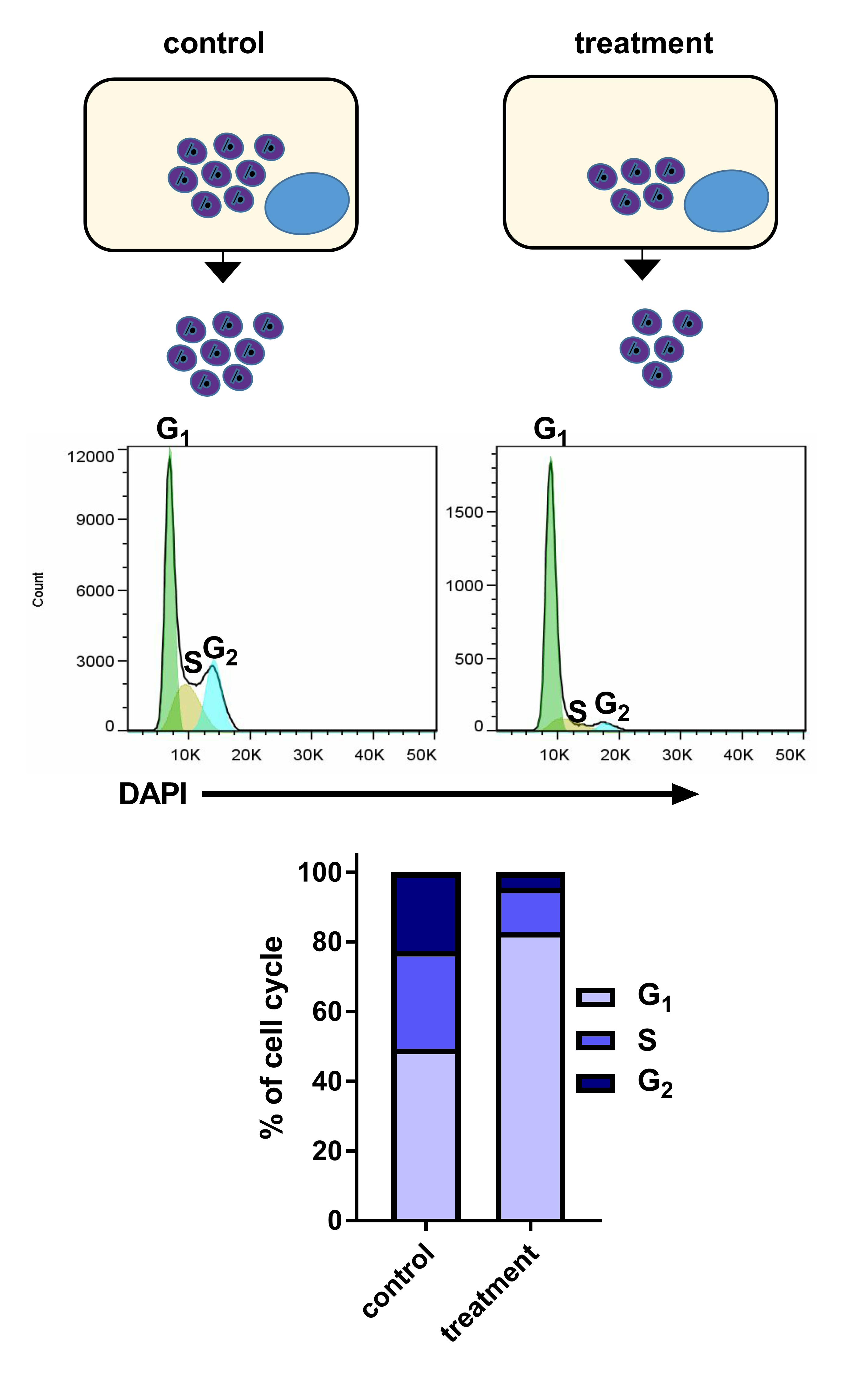

Supplement: FIG S2 [file mbo004183981sf2.tif]

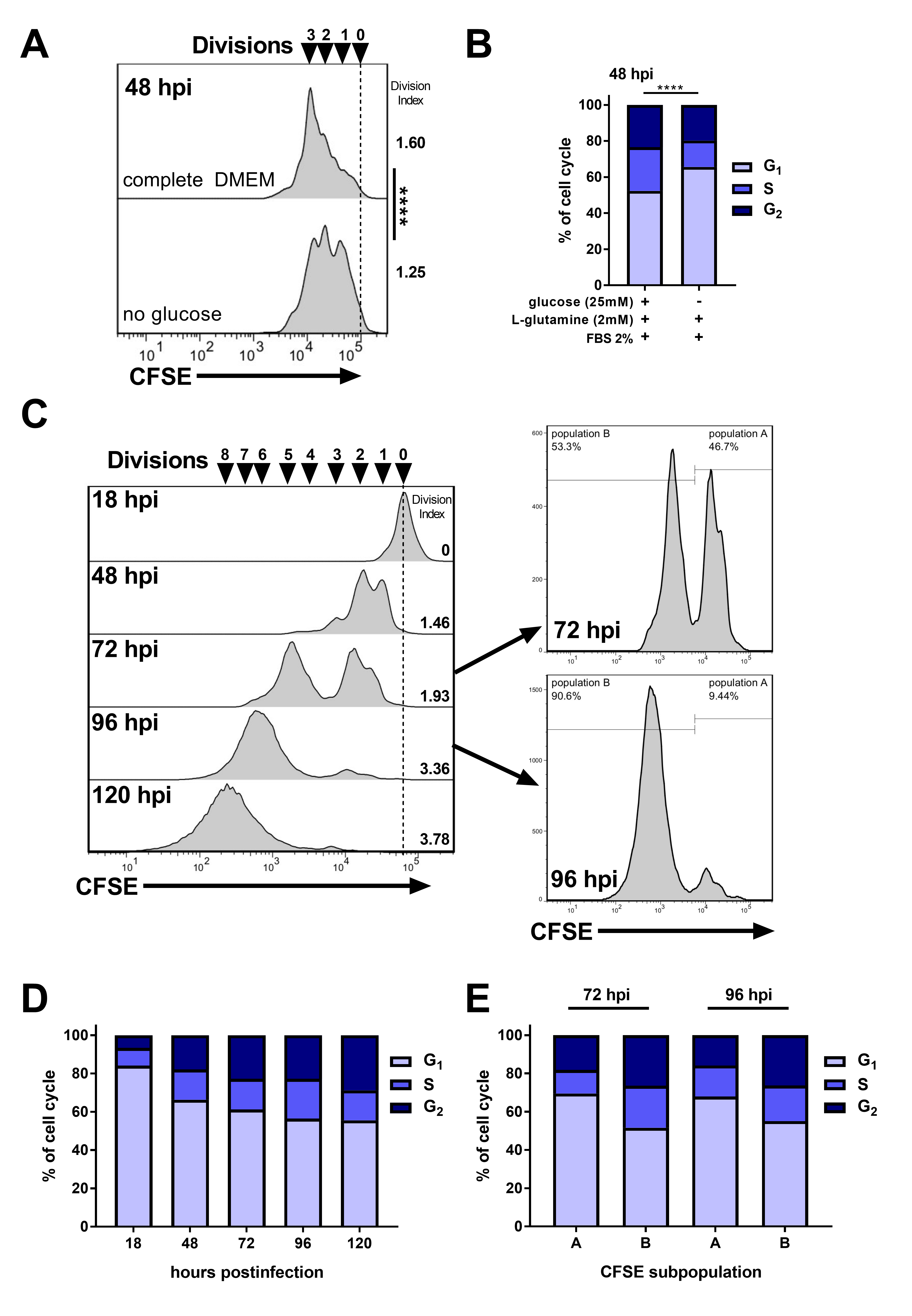

Supplement: FIG S3 [file mbo004183981sf3.tif]

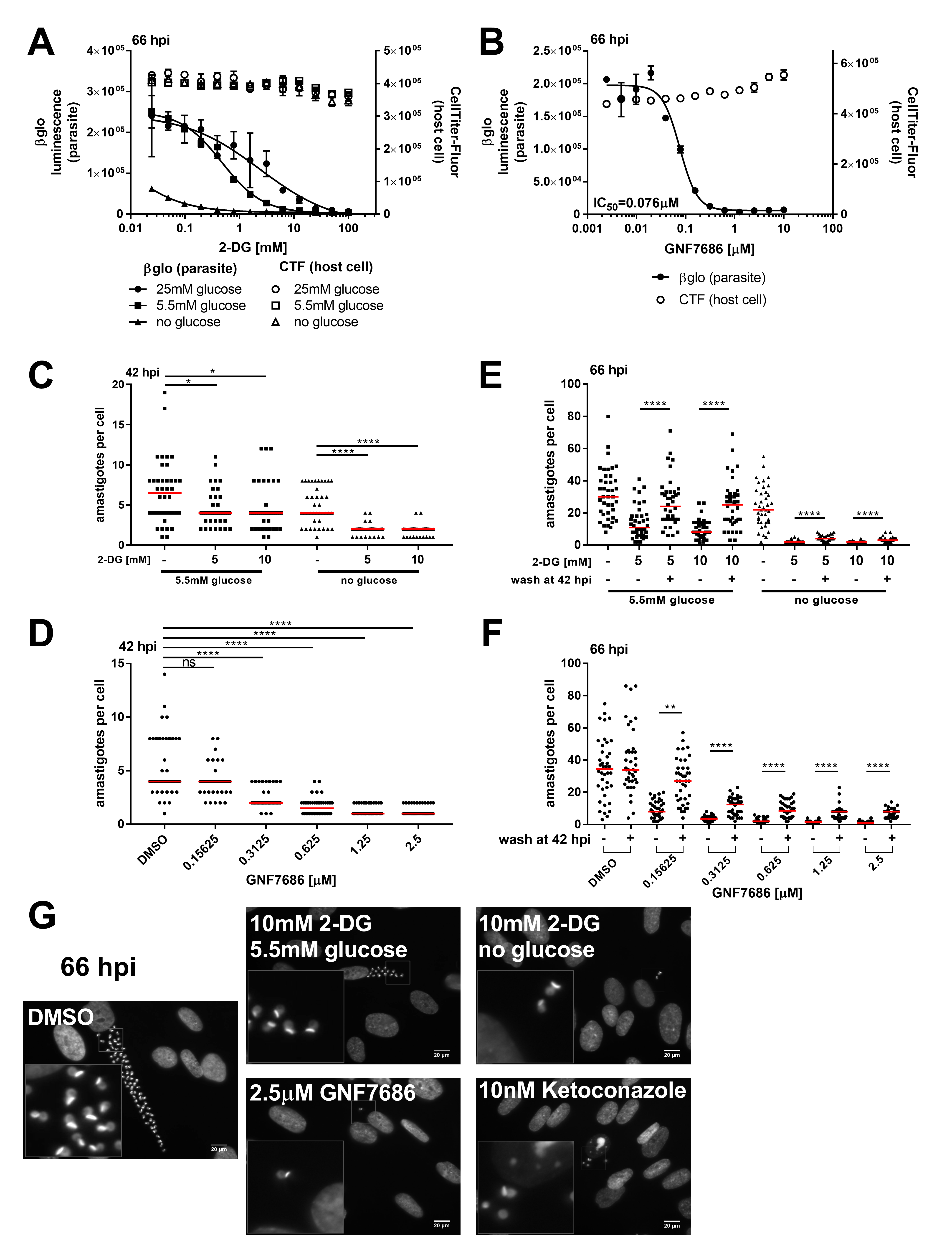

Supplement: FIG S4 [file mbo004183981sf4.tif]

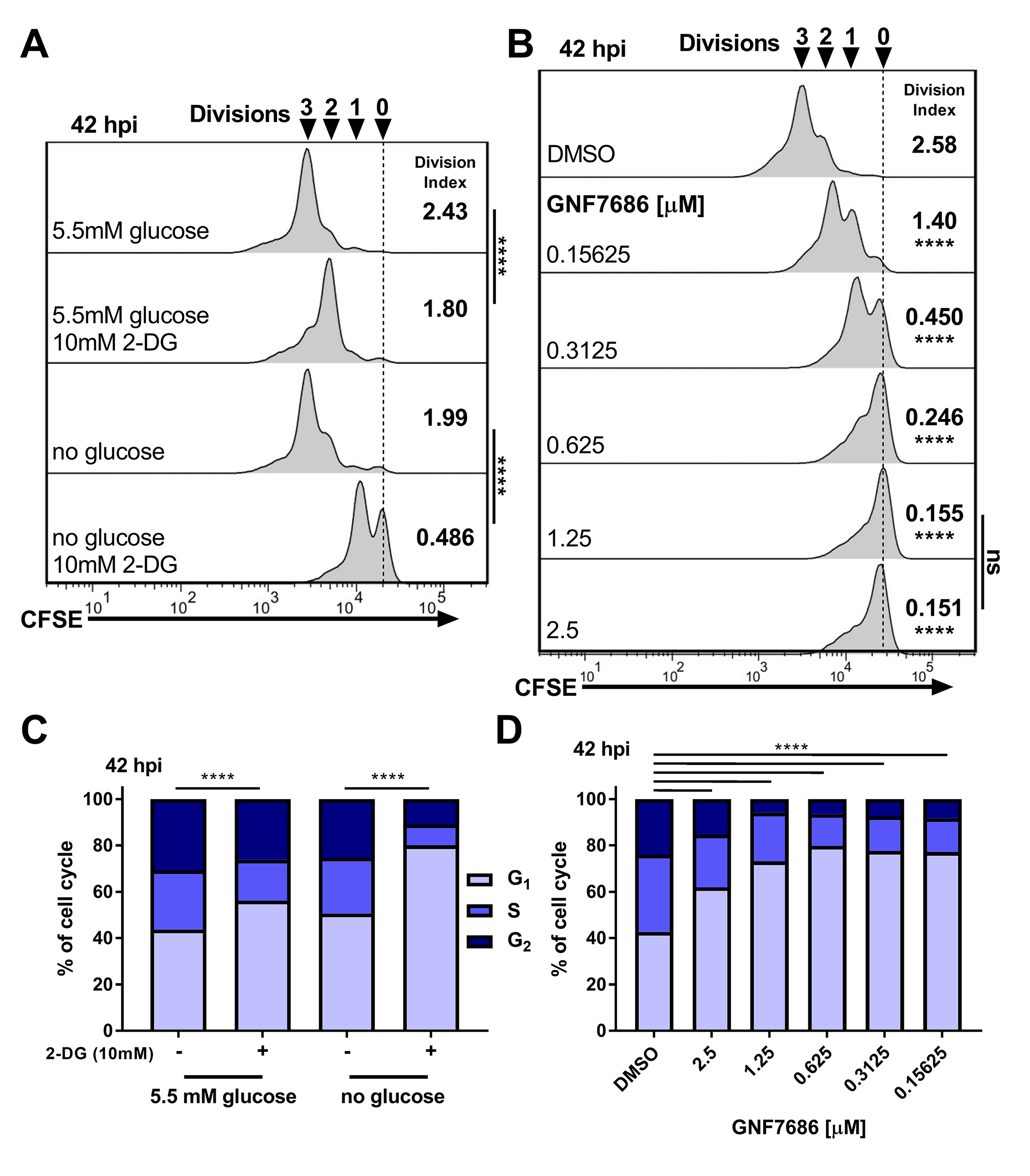

Supplement: FIG S5 [file mbo004183981sf5.tif]

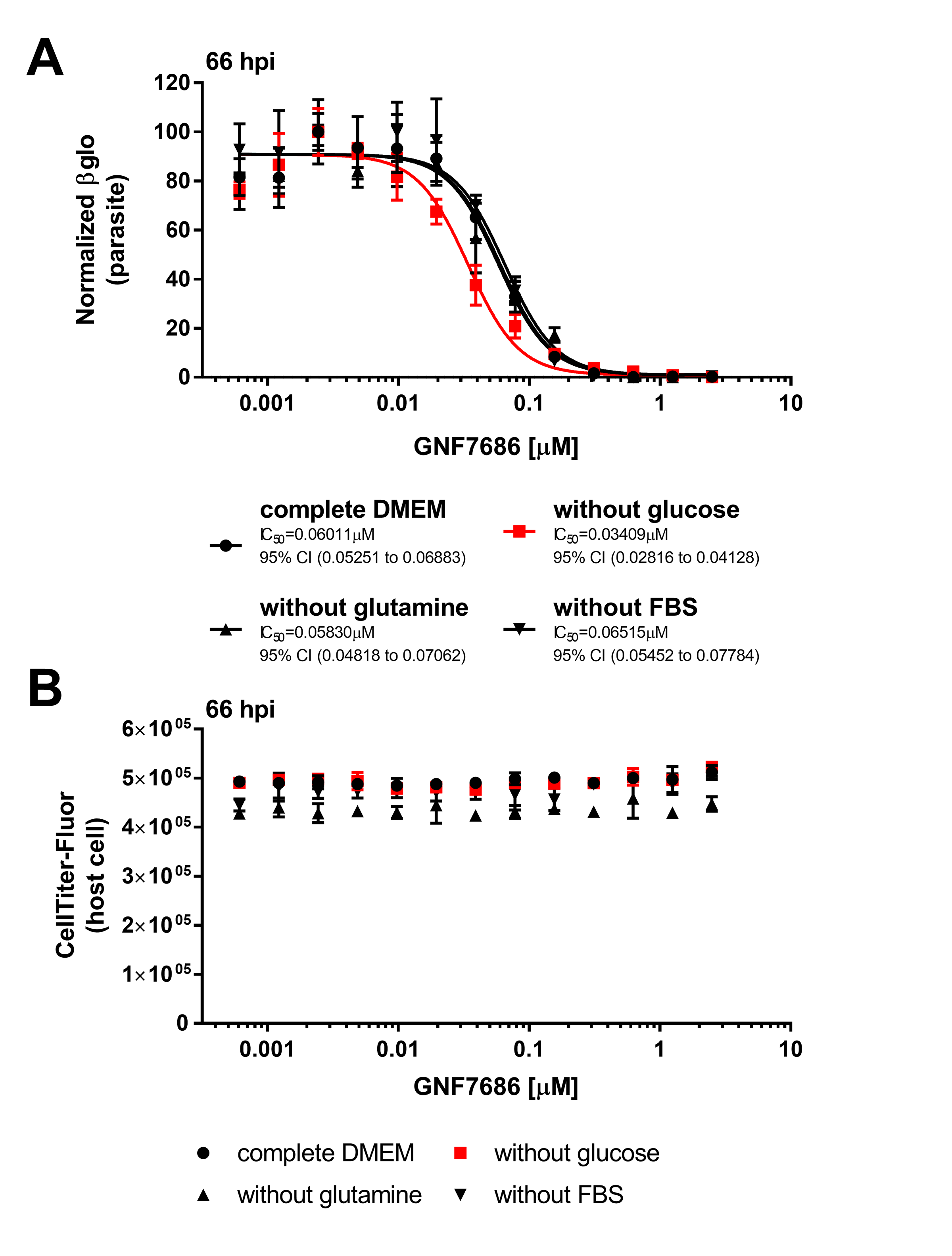

Supplement: FIG S6 [file mbo004183981sf6.tif]

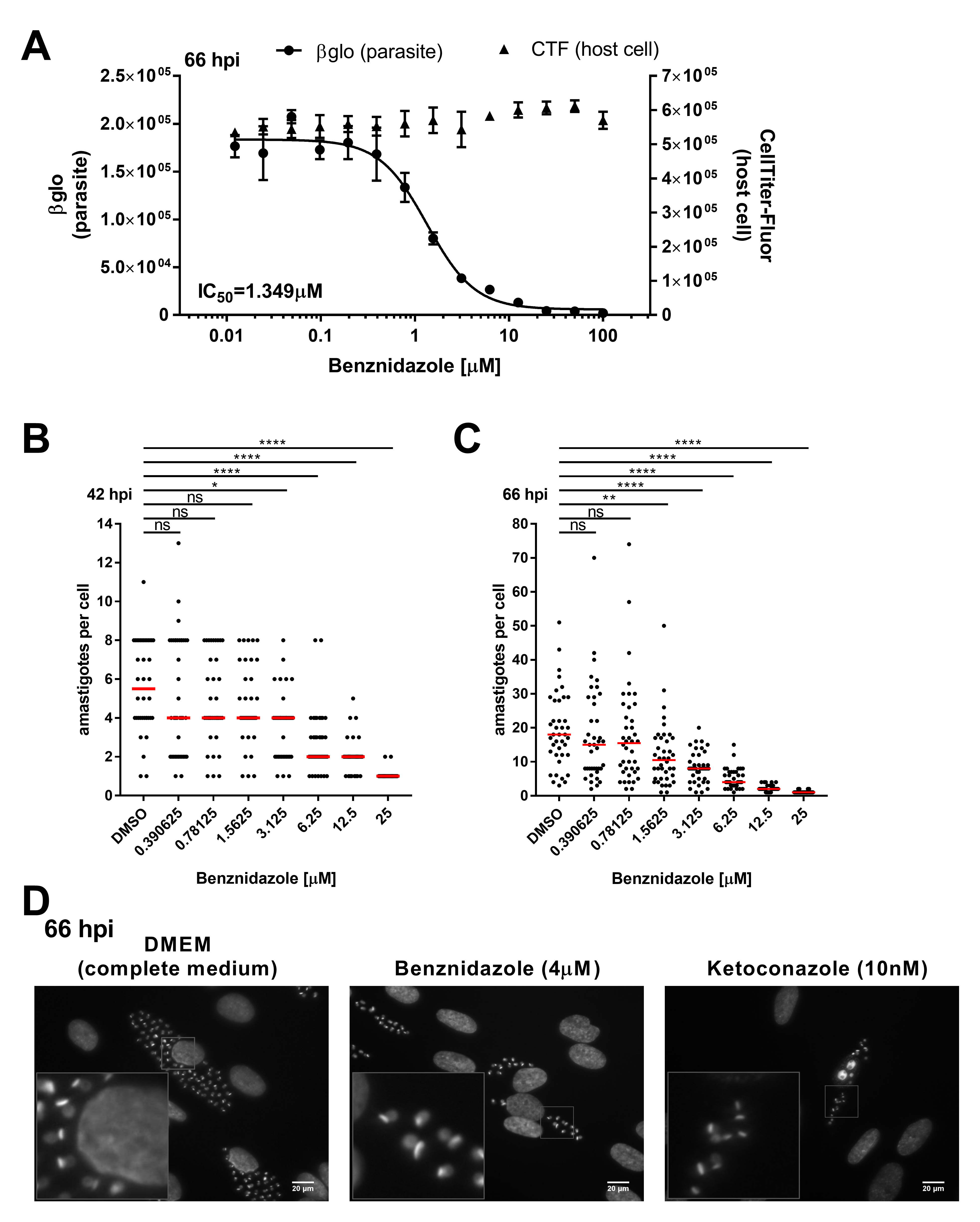

Supplement: FIG S7 [file mbo004183981sf7.tif]

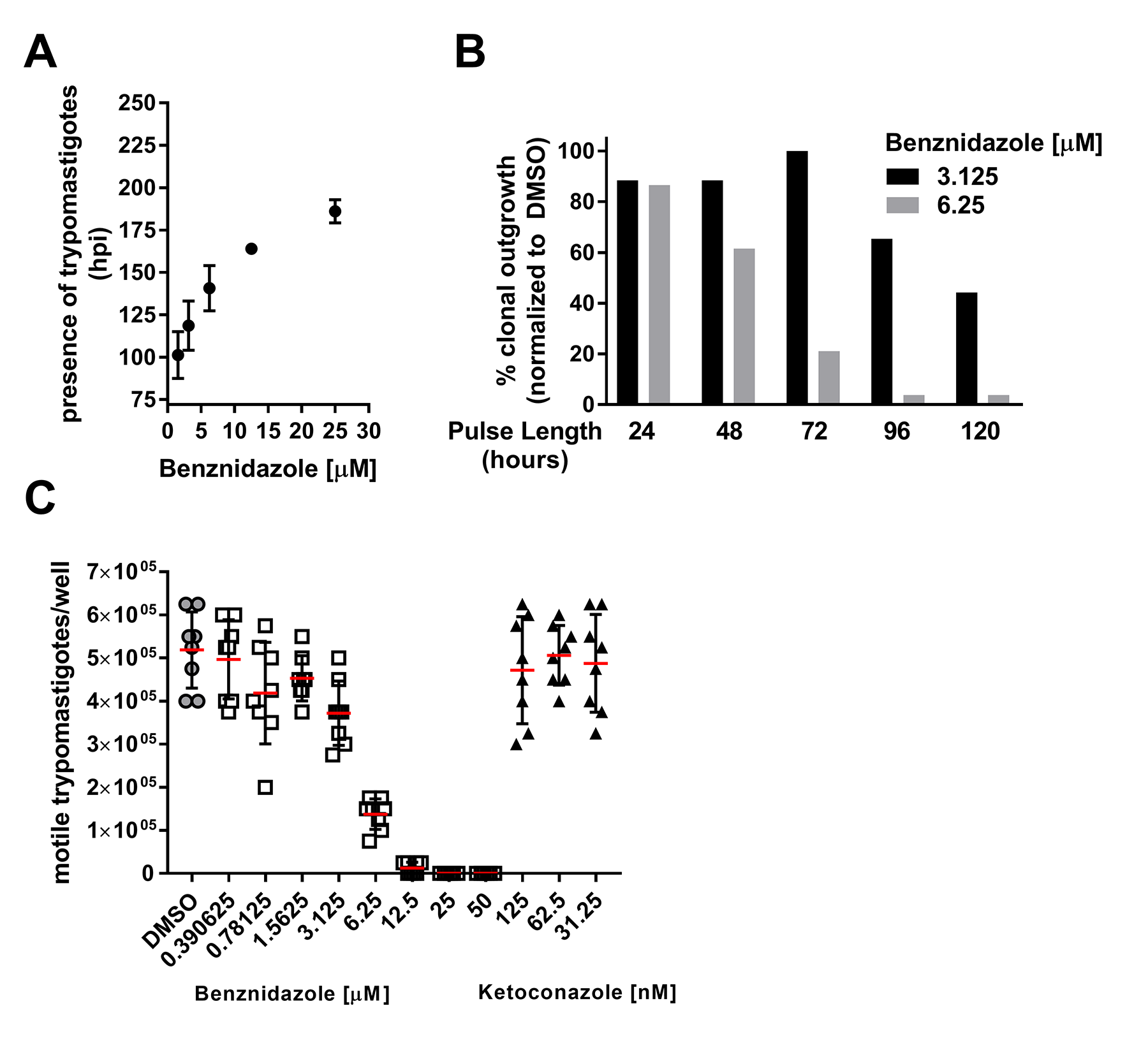

Supplement: FIG S8 [file mbo004183981sf8.tif]
